# Changes and continuities in gambling careers during the COVID-19 pandemic: a longitudinal qualitative study of regular sports bettors in Britain

**DOI:** 10.1186/s12889-024-21077-5

**Published:** 2025-01-07

**Authors:** Ashley Brown, Craig Donnachie, Nathan Critchlow, Christopher Bunn, Fiona Dobbie, Cindy M Gray, Richard Purves, Gerda Reith, Heather Wardle, Kate Hunt

**Affiliations:** 1 Institute for Social Marketing and Health, University of Stirling, Stirling, Scotland; 2 Department of Psychological Sciences and Health, University of Strathclyde, Glasgow, Scotland; 3 School of Social and Political Sciences, University of Glasgow, Glasgow, Scotland; 4 Usher Institute, University of Edinburgh, Edinburgh, Scotland

## Abstract

**Background:**

To explore continuities and changes in gambling behaviour during the COVID-19 pandemic and the factors that influenced these among a sample of regular sports bettors.

**Methods:**

A longitudinal qualitative study using in-depth interviews. Sixteen sports bettors living in Britain took part in the first interviews in July-November 2020, and 13 in the follow-up interviews in March-September 2021.

**Results:**

Individual patterns of gambling were episodic: it was common for gambling to increase during some periods of the pandemic and to decrease during others, reflecting the dynamic and (often) challenging circumstances which people were living through at the time. Changes and continuities in gambling during the pandemic were influenced by a range of factors which we have grouped into two main themes relating to ‘gambling and the sports landscape’ and ‘disruption to day-to-day life’. It was common for a constellation of factors to influence gambling behaviour rather than a single factor. These constellations of factors varied from person to person and at different times during the pandemic.

**Conclusions:**

Findings of the present study are consistent with earlier literature examining gambling careers before the advent of COVID-19 showing that gambling trajectories are non-linear. Our research suggests that ‘typical’ patterns of gambling behaviour (e.g. being episodic), and the broader known risk and protective factors within individuals, families, communities and societies have been amplified during the pandemic. Findings highlight the adaptability of the gambling industry to continue to reach consumers through product offerings and marketing even in a period of unprecedented restrictions on supply, and show the potential resulting harms of these actions among gamblers at risk of experiencing gambling problems. Taken together, findings from this study provide important new insights relevant to discussions about gambling regulation, and support calls for multifaceted and comprehensive policy, regulatory, and treatment approaches, to minimise gambling-related harms.

## Background

The global COVID-19 pandemic (hereafter ‘the pandemic’) triggered unprecedented disruption to everyday life. In Britain, a national lockdown was imposed on 23rd March 2020, which saw the closure of all gambling venues and the immediate suspension of almost all sporting events. Over the following two years further lockdowns and restrictions were implemented which impacted almost every aspect of everyday life.

People spent more time at home, sometimes isolated from family and friends during the COVID-19 pandemic. Prevalence of anxiety and depression increased worldwide [1] and people with the lowest incomes experienced the greatest financial losses compared with projected earnings before the pandemic [2]. Online gambling was readily available, and gambling operators took actions to maintain or grow their businesses (e.g. in relation to the marketing and promotion of gambling products), during a period of unique restrictions in supply. It is plausible that these changes resulted in increases in gambling and gambling harms during the COVID-19 pandemic given overlaps with known/potential risk factors [3–6]. Alternatively, decreased availability of certain gambling products/environments [7], and increased opportunities for self-reflection or engagement in alternative behaviours [8] during the COVID-19 pandemic could have resulted in decreased gambling and harms in some groups.

Recent reviews suggest that, while there had been an overall reduction in gambling participation during the pandemic, gambling increased among some sub-groups [9–12]. Being younger, male and having a higher problem gambling severity score was associated with increased gambling participation [9]. A rapid review [13] conducted in 2020 of 22 primary studies, which examined *reasons* for changes in gambling behaviour in the early stages of the pandemic, found that *decreased* gambling was driven by reduced availability of gambling products (because of closure of gambling venues and professional sports), reduced opportunities to participate in gambling due to disruptions to daily life, decreased motivation to gamble, and reductions in disposable income. The review noted that *increased* gambling during the pandemic was facilitated by feelings of boredom, stress and anxiety, changes in routines, improvements in finances and hopes of winning money to bolster earnings. Despite growing evidence about gambling behaviour during the pandemic, reviews have identified a paucity of qualitative research [10, 13] which could enrich understanding of how behaviour changed during the pandemic, and of the factors that could influence that change over time [10]. To our knowledge, only two (grey literature) publications have used qualitative methods to study gambling behaviour in Britain during the pandemic [14, 15]. A second evidence gap identified in reviews is the lack of longitudinal data collected beyond the first wave of restrictions imposed in 2020 in attempts to contain the pandemic [10]. This means that current evidence only provides a ‘snapshot’ of gambling behaviour, with longer term follow-up required to fully understand the implications of COVID-19 on gambling behaviour.

Existing longitudinal studies of gambling behaviour over time suggest that behaviour is highly fluid and can change in response to shifts in individual circumstances and wider social and environmental contexts. One of the first studies to explore this from a qualitative perspective was Reith and Dobbie’s [16] five year study of the ‘gambling careers’ of 50 ‘problem’ and ‘recreational’ gamblers in Scotland, UK. They found that gambling behaviour was highly changeable over even short periods of time, with changes influenced by a range of factors, particularly those affecting interpersonal relationships, employment and finances, and changes within participants’ local environment. Subsequent research that has adopted a similar methodological and conceptual ‘careers’ approach has also highlighted the dynamic nature of gambling behaviour and ways in which it changed in relation to shifts in gamblers’ social, financial and environmental contexts [17, 18].

The current study seeks to follow Reith and Dobbie’s [16] concept of gambling ‘careers’ and presents findings from a longitudinal analysis of qualitative interviews with regular sports bettors in Britain. Data reported here were collected during two different periods during the first 18 months of the pandemic, in July-November 2020 and April-September 2021. Our objective was to explore continuities and changes in gambling during the pandemic among a sample of regular sports bettors and the factors that influenced these. The importance of remaining ‘vigilant’ to the potential harms occurring in some groups of gamblers during the pandemic has been highlighted [7, 10, 12]. Understanding how consumers responded to an unprecedented restriction on some forms of gambling during the pandemic can provide important insights to inform ongoing discussions about gambling regulations, including, in the UK, the regulatory changes proposed in the Gambling Act Review White Paper in areas such as marketing and advertising.

## Methods

### Study context

In this section, we provide an overview of ‘lockdown’ measures to control the spread of the COVID-19 virus implemented in England and Scotland, the nations in which participants in this study resided, focusing on events in the lead up to and around the time of the interviews. In the initial lockdown commencing 23rd March 2020, people were required to stay at home except for ‘essential’ activities (e.g. shopping for necessities, daily exercise, providing care, travelling to and from work in essential occupations, and meeting medical needs) [19–21]. During this lockdown period, which lasted nationally for approximately three months, almost all professional sports events [22] were cancelled and non-essential businesses, including gambling premises such as bookmakers, casinos, arcades and bingo halls, were required to close.

During the early stages of the pandemic, the Betting and Gaming Council (BGC) in Britain introduced a voluntary action plan establishing ‘the standards expected of…members during the COVID-19 pandemic’ [23]. This covered areas such as safer gambling messages, deposit limits, signposting customers to help and support, and intervening if gambling behaviour escalated ‘beyond normal pre-pandemic patterns’. The BGC also committed to voluntarily remove all TV and radio gambling advertising during the spring 2020 lockdown [24].

Timelines and approaches for reopening society after the initial lockdown varied across Scotland and England. Broadly, restrictions on leaving home and opening businesses and other settings gradually eased from June 2020. The first European major football league to return was the Bundesliga (Germany) in May 2020. Several weeks later the English Premier League Football restarted, initially without in-stadia spectators. Restrictions on the opening of some gambling premises began to ease from June 2020.

Some infection control measures were subsequently reimposed at local or national level. Further national lockdowns were in place in England from November 2020, and again from January 2021. From January 2021, all of mainland Scotland was in the highest level of lockdown (level 5). Professional sport continued during the 2020–2021 winter lockdowns in England and Scotland, but gambling venues were closed. In England and Scotland, lockdown restrictions began to be eased from April 2021. This included the gradual reopening of gambling venues, with some measures to control the spread of the virus in place (e.g. limiting the number of customers and no streaming of live sports), and the return of spectators to live sporting events [25].

### Sampling and recruitment

Data are from the multi-method, longitudinal ‘Betting and Gaming COVID-19 Impact Study’ [26], which was designed to examine how the pandemic impacted on two groups, ‘regular’ sports bettors and young people, during 2020 and 2021. This paper reports findings of qualitative interviews with regular sports bettors; analyses of survey and marketing data from other strands of the research are reported elsewhere [5, 7, 26–28]. Ethical approval for the qualitative element of the study was granted by the University of Stirling’s General University Ethics Panel (ref: GUEP (19 20) 930).

Sports bettors were chosen as a group which might be particularly impacted by the pandemic for several reasons, including: significant changes in the sports and gambling landscapes following restrictions in Britain; the potential for increased industry marketing post-reopening; and the heightened risk of many sports gamblers [26]. Longitudinal one-to-one qualitative interviews were chosen as they enable in-depth and confidential exploration of views, experiences and behaviours and whether, how and why these may change over time. Repeated interviews with individuals, 9–14 months apart were selected as a means of capturing continuities and change over the first 18 months of the pandemic.

Sixteen sports bettors took part in the first telephone interviews in July-November 2020, and 13 in the follow-up interviews in March-September 2021. There was no clear pattern in sample attrition; the three participants who did not take part in a second interview had different risks of gambling harms as assessed by a screening instrument at baseline. Target sample sizes were set in advance of fieldwork, informed by practical considerations and judgements about how many interviews might be required to capture sufficient diversity. All participants were aged 18+, resident in Britain, and had bet on sports at least once a month before the initial COVID-19 lockdown. We used multiple strategies to recruit participants: social media (Twitter and Facebook), an online portal at the study’s host university, and snowballing. Participants were offered a £20 online shopping voucher as a gesture of thanks for undertaking an interview.

We monitored key participant characteristics (age, sex, mode of sport betting) during recruitment to achieve as diverse a sample as possible. At the end of interviews, participants completed the 3-item ‘Problem Gambling Severity Index’ (PGSI) [29] with each item scored on a four-point scale (0 = Never to 3 = Almost always), to help classify participants in respect of the severity of their gambling behaviour at that point in time, and monitor major movement across categories between the first and second interview. A score of 0 represents someone not experiencing any issues included in the PGSI scale. A score of 1 represents those at low risk of experiencing gambling problems. A score of 2 or 3 represents those at moderate risk of experiencing gambling problems. A score of 4 or greater (to the maximum of 9) represents those experiencing gambling problems. We use this terminology (‘no risk’, ‘low risk’, ‘moderate risk’, and ‘experiencing problems’) in the remainer of the paper whenever referring back to the PGSI score of a participant at the time of the interview.

### Data collection

Prior to telephone interviews, participants provided written or audio-recorded consent. Interviews were conducted by experienced qualitative researchers (CD, AB, RP and FD) with the aid of a topic guide developed to reflect the study objectives, emerging evidence and subject expertise within the research team. The topic guide used in 2020 covered participant background and life circumstances, gambling behaviours before and during the first lockdown (~ March-June 2020), and gambling advertising and marketing. The topic guide was updated in 2021 to cover gambling behaviour during subsequent periods of the pandemic. Topic guides were used flexibly by adjusting question wording and topic order as appropriate and encouraging participants to raise any additional points they considered important.

### Analysis

Interviews were audio-recorded and fully transcribed with participant permission. Transcripts were de-identified and thematically analysed with the aid of the Framework approach [30], to support systematic, comprehensive and auditable analysis of rich and often unwieldy data [31]. First, we coded and summarised data under broad themes using a ‘thematic framework’ developed using both inductive and deductive techniques (e.g. reading transcripts and literature and discussion among the authorship team). Summarised data were displayed in a grid (row = transcript, column = theme) in NVivo 12 (QSR), to support both theme and case-based analysis. Second, extracts from interviews and summarised data relating to themes relevant to this paper were examined in detail by AB to identify the range and diversity of views and experiences, possible links across the data, and to create emerging themes. Third, themes were developed over multiple iterations and refined in response to re-examining data, and discussion and feedback from the wider authorship team: CD, CB, RP and KH read a sample of transcripts; other authors contributed methodological and subject expertise. The final interpretation of the data was agreed by all authors.

Results presented here are based on analysis of the full dataset (29 interviews), unless otherwise stated. Continuities and changes in gambling behaviour were identified based on examining participant accounts and PGSI scores, supported by displaying data in a Framework grid. Illustrative quotes for each theme are included, with participant serial number, year of interview, and PGSI category at the time of interview (NPG = someone not experiencing any issues included in the PGSI scale), LRG = someone who is at low risk of experiencing gambling problems, MRG = someone who is at medium risk of experiencing gambling problems and PG = someone who is experiencing gambling problems).

## Results

Table 1 shows key characteristics of the achieved sample in 2020 and 2021.

**Table 1:** Sample characteristics

		2020	2021
Sex	Male	13	10
	Female	3	3
Age group	18–30	7	5
	31–40	4	4
	41–50	3	2
	51+	2	2
Ethnicity	White ethnic group	15	12
	Other ethnic group	1	1
Education	University degree No University degree	9 7	7 6
PGSI Score	Non-problem gambling	7	7
	Low risk gambling	1	0
	Medium risk gambling	5	4
	Problem gambling	3	2

While PGSI scores are indictive of problem gambling severity, misclassifications can occur [32]. For example, in this sample one participant who scored ‘0’ on the PGSI described recurring problems with gambling (e.g. difficulties controlling gambling behaviour) and some negative consequences of gambling (e.g. financial losses) during the interview. This highlights the importance of participant accounts in providing a more complete understanding of gambling behaviour.

The most common pre-pandemic sports betting activities mentioned were gambling on football and horse racing. Several participants also participated in other forms of gambling pre-pandemic such as casino gambling, lottery, scratch cards, and fixed odds betting terminals.

The pandemic affected participants’ gambling behaviour in a number of ways and, as others have reported in non-COVID-19 times [16], changes in gambling behaviour and people’s gambling ‘careers’ varied between individuals and over time. Fluctuations in levels of gambling on one or more activities were frequently discussed in participant accounts: it was common for there to be times during the pandemic when an individual described increased gambling and other periods when they said that their gambling decreased. Thus, participant accounts highlighted the fluidity of gambling behaviour in the context of the dynamic circumstances in which people were living during the pandemic. Despite this, only three participants were re-classified into different PGSI categories between the initial and follow-up interviews (one participant’s PGSI classification increased in severity and two decreased).

In the next sections, we describe and explain continuities and changes in gambling behaviour during the pandemic in more detail. We have grouped factors into two distinct, but interrelated themes identified from the accounts of our sample of regular sports bettors: ‘changes to the gambling and the sports landscape’ and ‘COVID-19’s impact on day-to-day life’.

### Changes to the gambling and sports landscape

#### Disruption to the sporting calendar and sports betting opportunities

Participants frequently reported stopping gambling altogether or decreased gambling around the period of major sport shutdown during the initial ‘lockdown’ in spring 2020 across the UK and Europe (*01-2020-NPG: I just don’t remember betting at all [during the early lockdown] …There was nothing to bet on*). However, all of those who reported stopping at this time had resumed gambling by the time of the first interview.

These reductions reflected some people’s reduced desire and interest to gamble on the limited opportunities that were available, or decreased desire to gamble in general:

*04-2020-NPG: …to begin with*,* I was barely gambling at all during lockdown because there was nothing I was looking at that was worth betting on*,* because it was all like Indian cricket and stuff like that*,* stuff I have no idea over.*

By contrast, other people (more often those at moderate risk of experiencing gambling problems or experiencing gambling problems) reported increased gambling and/or having concerns about gambling when major sport was suspended:

***Int: during this initial lockdown…did you ever have any concerns about your gambling?***

*06-2020-NPG: A bit*,* yes. [I was gambling] normally every day and then realising*,* ‘hang on a minute*,* what am I doing?’ It’s only until you’re losing loads of money that you’re like*,* ‘…maybe I should just calm down a bit.’*

The shutdown of major sports also contributed to an uptake of new sports betting activities by some participants, across PGSI groups. Examples included betting on different sports fixtures (such as Belarusian football league) or betting on new sports/e-sports activities (such as e-motor racing). Uptake of new sports betting activities was often temporary while more stringent COVID-19 restrictions were in place.

*15-2020-NPG: We did find something [to bet on]. And actually this is pretty good*,* you could make this pay. On Sundays there were e-grand prix*,* sort of they’d have grand prix*,* with formula one drivers in simulators. And*,* one or two of the firms started betting on it*,* but they did not have a clue*,* they had no idea how*,* they literally were guessing at pricing up.*

The return of ‘mainstream’ sport (such as English Premier League football and resumption of horse racing) in summer 2020 resulted in further changes in participants’ gambling activities as they were able to resume sports betting activities. For some participants, across PGSI groups, the return of sport after a period of major disruption provoked feelings of novelty, excitement and a heightened desire to gamble, which contributed to increased gambling:

*16-2020-LRG: I think in the first couple of weeks the excitement of having the footie back and you just think “Right*,* let’s go for this*,* let’s go hell for leather!”*

The novelty of being able to watch and bet on sports again led some participants to bet on fixtures they would not usually bet on, even if they ‘*didn’t care about the outcome*’(16-2020-LRG).

Changes in fixture scheduling during the pandemic, particularly in relation to football and horse racing which were two of the most popular sports to bet on amongst our participants, could lead to increases in gambling. Specifically, participants across PGSI groups explained how the compressed sports calendar created a plethora of opportunities for watching and betting on sports, which were less restricted to set days of the week and times of year as had been the case pre-pandemic:

*10-2020-NPG: It went from famine to feast really*,* so there’s been loads of horse racing now*,* to watch*,* so it’s quite intense*,* following it at the moment.*

*05-2021-NPG: there were something almost every single day for a while*,* because…of the way that seasons were truncated*,* and then squashed together*,* and now everything’s been squashed together for the [football] World Cup coming up in 2022 and the Euros*,* there’s like football Monday*,* Tuesday*,* Wednesday*,* Thursday*,* Friday*,* Saturday*,* Sunday. You kind of almost don’t get a chance to have a break from it.*

Other ways in which disruptions to the sporting calendar during the pandemic contributed to increased gambling included high numbers of sports events being televised, which was particularly significant for those for whom betting was bound up with watching a live event:

*11-2021-MRG: I think the fact that you could watch all but essentially every single game*,* so the fact that people like myself…[who] only wants to gamble on what they can watch*,* logically that can increase the amount that I was gambling.*

COVID-19 restrictions were also perceived to produce favourable betting odds for some fixtures e.g. because bookmakers struggled to account for any impact of the loss of live spectators in their setting of the odds.

*11-2020-MRG: [there are] new opportunities in terms of like a chance to win money in ways that have not been won before because even the bookmakers are struggling to kind of figure out their algorithms for it [football fixtures].*

However, disruptions to the sporting calendar, after the reopening of sport, could also contribute to decreases in gambling. For example, a participant who was not experiencing gambling problems explained how his gambling reduced once the ‘novelty’ of being able to bet on sports again had subsided:

*05-2021-NPG: I…would have put on a few more bets because there was just more football happening*,* and it was just like day after day after day. But then again*,* that was just for a couple of weeks…it’s probably [because] the novelty wore off.*

Another participant (13-2020-PG) who was experiencing gambling problems described a ‘*massive disinclination*’ to gamble when games were played behind ‘closed doors’: ‘*it was just a massive turnoff*.’

#### Closure (and reopening) of gambling premises and switching to online gambling

Participants, particularly those who had primarily participated in land-based gambling pre-pandemic, reported switching during the height of pandemic restrictions to online gambling products (such as online casino, online scratch cards and online bingo) or using online gambling products more than usual ‘*because the stores were closed*’ (08-2020-PG).

This increase in online gambling was associated with an overall increase in gambling activity for some participants. Several reasons were mentioned including the ease and convenience of online gambling, the ability to gamble anytime online, and having ready access to an extensive number of gambling products online (e.g. international sports events) while spending more time at home.

*02-2020-PG: …my betting didn’t used to be as bad before the COVID*,* but see since March*,* when the [betting] shops closed*,* that’s when I went off the rails because I knew that I could just go online*,* just put money in my account*,* and just bet whatever I want…*.

The reopening of gambling premises provided renewed opportunities for participation in land-based gambling. For example, one participant said they were ‘*much more likely to have a bet in a race*’ when they were able to attend the racecourse and see the horses (10-2021-NPG). However, the perceived lack of atmosphere in betting shops (because of continuing COVID-19 restrictions) was a disincentive to others:

*08-2021-MRG: So with the betting shops [before] COVID-19*,* things were good*,* but now there’s no one in a betting shop*,* there’s no game to play. It’s just gone downhill*,* like what is there to play*,* what is there to see…there’s no point in a betting shop.*

No participants reported having fully returned to their pre-pandemic participation in land-based gambling because of factors such as ongoing restrictions during the fieldwork period, reduced appeal of land-based gambling during the pandemic and changes in personal circumstances.

#### Exposure to gambling marketing

Participants described varied levels of awareness of gambling marketing and differential impacts of exposures to marketing on emotions and behaviour during the pandemic. Among those not experiencing gambling problems, participants often said that they did not take a great deal of notice of marketing, and generally did not mention a negative influence on their behaviour.

In contrast, among those at moderate risk of experiencing gambling problems or experiencing gambling problems, some described feeling inundated by marketing, particularly direct marketing and promotions, during the pandemic, reportedly leading to harms. This included marketing from operators who they presumed were not licensed in Britain. High awareness of gambling marketing was reported by some people at risk of, or experiencing, gambling problems during the first lockdown, and, for some, awareness of marketing continued to be similarly high or higher in subsequent phases of the pandemic, reportedly contributing to further periods of increased gambling.

One participant (14-2021-PG) who was experiencing gambling problems highlighted how hard it was to self-manage their gambling behaviour in the context of reportedly predatory (‘*they know that….[gambling] is a weakness of mine*’) direct marketing during the pandemic. He went on to describe how, despite his considerable efforts to make it difficult or impossible for him to gamble, he ended up gambling again for a while in response to marketing texts:

*14-2021-PG: I just think it was one text message too much.…I suppose if you tap away at a tree with a knife*,* a small knife*,* it doesn’t chop down at first but if you chop enough at it eventually you’re going to get the outcome you want.*

It was evident that sometimes the nature of gambling marketing could be distressing, when participants experienced marketing as excessive or aggressive. For example, one participant who was experiencing gambling problems felt ‘*pressure*’ to gamble because of the ‘*shocking*’ volume of marketing sent to them by gambling companies (02-2021-PG), and another said:

*08-2020-PG: It did bother me because I thought to myself*,* ‘I’m constantly being bombarded with emails with marketing information about betting and gambling’.*

Promotions for online gambling (e.g. free spins or free bets), seen via direct marketing, online advertising or TV, were reported to have contributed to an escalation in gambling among some people at moderate risk of experiencing gambling problems or who were experiencing gambling problems. The man quoted earlier in relation to the switch from land-based to online gambling described how it was a free bets promotion that enticed him to open a new online account (because bookmakers were closed). This resulted in his gambling “*going off the rails*” because he deposited more money than he planned to spend to qualify for offers, and then tried to chase his losses:

*02-2020-PG: I put the £10 on. I put that on a horse and then I put the three bets on something silly like virtual racing or something*,* but when I lost that…I used to say to my partner “I’m going to put more money on”. She went*,* “No!”. She tried to put me off by saying “You’ve already had two free bets…” and I used to sneak away and put money into my account…*.

The risks in being able to act immediately on gambling cues within marketing and promotions in the context of having to spend more time at home and feeling bored (see below) during the pandemic were well articulated in some participants’ accounts.

*14-2020-MRG: “I’m not making excuses for myself*,* I kept seeing adverts*,* I kept…and one evening was just terribly bored*,* opened [online] account up with [gambling company] and*,* yeah*,* just sat there and barely even…[I] went to work with about three hours’ sleep because before I knew it was two o’clock in the morning*,* yeah*,* just because think it was constantly in front of me.*

Some people who were not experiencing gambling problems or who were at lower risk of experiencing gambling problems said that exposures to gambling marketing while watching live sports events on TV, or receiving mobile phone notifications from gambling companies, had prompted increased interest and participation in sports betting on occasions during the pandemic. For example, participant (16-2020-LRG) reported an ‘uplift’ in his gambling because ‘*every other advert [was] a betting advert’* immediately following the return of live sports events.

In contrast, not being exposed to as much gambling marketing and gambling cues as usual during the initial lockdown, because of changes in his daily routine, helped another participant experiencing gambling problems to cease gambling for a period.

In some cases, gambling marketing and promotions influenced individuals to start new gambling activities during the pandemic, sometimes contributing to higher gambling participation:

*09-2021-MRG: [I participated in] online bingo. Which I decided to try*,* because there was*,* you know*,* I think*,* free…five free cards of bingo or something like that*,* so I decided to try it.*

### COVID-19’s impact on day-to-day life

The pandemic caused major disruption to day-to-day life, which influenced changes in some people’s levels of gambling, particularly during the initial 2020 lockdown when sporting events were suspended and to a lesser extent during subsequent lockdowns in 2020 and 2021, during which sporting events continued.

#### Work and occupying time

Some participants across PGSI groups described how disruptions (such as having few ways to occupy time, being out of work or ‘furloughed’ or having reduced work) had contributed to increased gambling:

*09-2021-MRG: A lot of the reason [for gambling] was because I was stuck inside*,* and it was winter*,* there was nothing to do*,* it was lockdown.*

*07-2021-MRG: But through the summer it was kind of extensive because there wasn’t anything else to do at all*,* so it kind of got a bit bigger at the end compared to what it had been.*

Spending more time at home during the pandemic had enabled some people to (markedly) increase time spent watching and betting on sports:

*01-2020-NPG: I have more time to spend on doing race cards and what have you. Before the lockdown*,* I would sort of scramble off maybe one card before I had to go and do something else. I’ve had a lot more time to sit around.*

Some people reported that their gambling decreased after resuming parts of their usual routines during periods when COVID-19 restrictions eased and they consequently had less time or inclination to watch sport and gamble.

*12-2020-MRG: I have got a lot going on in my life*,* in terms of*,* I do have a lot of hobbies*,* and I’ve got a lot of work*,* and do go to the gym and stuff*,* so I don’t really need to fill my time up with things like that. So*,* now we’ve got more things to do*,* I don’t really fill my time doing it*,* to be honest.*

Similarly, gambling also sometimes decreased following important life changes which occurred during the pandemic, such as becoming employed or changing jobs.

*10-2021-NPG: When I went back to work [after a period not working in the early pandemic] it [gambling] became less because obviously I now can’t watch as much [horse racing] so*,* I would say*,* it’s probably been a bit less since then…my new job has been pretty busy and a bit sort of like*,* well*,* I’ve got enough to be dealing with today. I haven’t got enough head space to*,* sort of…you know*,* or time to look at the races.*

#### Gambling emotions, and coping strategies

Increased gambling was also influenced by negative emotional responses to the pandemic and associated disruptions to daily life. Boredom was seen as being an important precipitator:

*03-2021-NPG: There probably has been a lot more boring weeks than before. It [gambling] probably has been a wee bit more.*

Low mood and stress related to the course of, or reactions to, the pandemic were also mentioned as factors which sometimes contributed to increased gambling during this time:

*02-2021-PG: I don’t like being off work*,* I’ve got to be at work all the time…I just couldn’t hack the lockdown*,* for some apparent reason. And that’s when I [used] my online betting app. I just*,* I was just betting silly amounts of money.*

A participant at low risk of experiencing gambling problems described how a period of increased gambling during the initial lockdown was linked to feelings of having fewer professional and social responsibilities because of COVID-19 related changes to his routines. These responsibilities had previously helped to curb his gambling:

*16-2020-LRG: …that first month [of lockdown] where everything has changed*,* everything was completely different and it almost felt like I didn’t have any responsibility because work was drying up*,* it didn’t feel like I had any responsibility to my girlfriend necessarily*,* because I’d talk to her and it would make me feel better about certain things*,* but I wasn’t seeing her. I wasn’t seeing my family or anything like that*,* so it felt like “strip everything away and just do what you want to do!”.*

For some people who gambled more during various stages of the pandemic in 2020 and 2021, sport and gambling were, at times, associated with positive feelings, such as anticipation, excitement, pleasure, relaxation and escapism, and so appeared to be used as a means of coping with disruption caused by COVID-19 restrictions or filling time:

*01-2021-NPG: I wonder whether I’d have felt much more adrift without the horseracing on TV….in that it gave me something to do. Something to do in the morning first thing and then throughout the afternoon. So it took up…it takes up quite a bit of my time. So it filled the gap for me I think.*

Restrictions to activities outside of the home during pandemic restrictions sometimes removed the option to engage in alternative coping strategies or displacement activities:

*14-2021-PG: So*,* yeah*,* I mean*,* like I said*,* little things like the gym not being open anymore. The gym is good for me as well because an hour and a half of my time where nothing else in the world matters. You’re in the gym*,* it could be anywhere*,* an hour and a half goes like that in a gym.*

Conversely, the disruption to day-to-day life caused by the pandemic also sometimes contributed to decreased gambling when participants found other ways to occupy time such as gardening, physical activity or spending time with a partner.

*13-2020-PG: …it’s…because it [pandemic] restricted my choices I had do you think*,* “Right*,* what else can I do? How else can I get my kicks?” And I found I did it through exercise.*

#### Social networks

Social networks were a contributory factor in some accounts of gambling change during the pandemic. Some younger participants described a greater reliance on gambling for social connection during the pandemic, which contributed to increased gambling:

*12-2020-MRG ….was playing quite a bit of poker as well*,* cause that was the only thing that you could really do with your friends*,* like*,* you could all join the same sort of games online….*

Social influence from online gambling communities or family members could encourage increased gambling, or uptake of new gambling activities:

*09-2021-MRG: my brother started to bet on horseracing*,* and then I had some free bets for horseracing*,* so I started doing that. And I used to do it probably like every other day*,* just because there were races on all the time.*

Social isolation, alongside boredom as discussed earlier, was another contributory factor to increased gambling:

*09-2021-MRG: sometimes I just get a bit self-isolated and bored by myself*,* that’s when…because it passes the time*,* I do it on the computer.*

In contrast, spending less time with peers in gambling environments (e.g. pubs where football games are being shown) or changes in social networks could lead to decreased gambling:

*03-2020-NPG: I don’t see my friends as often*,* so I think that’s what probably drove it down as well a bit. Like*,* I imagine when the lockdown lifts…’cause I’ve been living away from [city] where most of my friends are*,* even then I still haven’t seen them much. But I think if I was sitting in the pub with them on a Saturday or whatever day and there was football on*,* I’d probably end up putting a bet on.*

#### Financial circumstances

Changes in people’s financial circumstances could influence their gambling behaviour during the pandemic, across PGSI groups. Some people’s gambling increased following improvements in financial circumstances (e.g. getting a pay increase or a windfall) or reductions in outgoings due to changes in day-to-day life and not being able to go out due to the pandemic.

*11-2021-MRG: I would say it [gambling] probably went up during the pandemic…I think my gross spending on frivolities*,* for want of a better word*,* probably went up in general I think because of the fact I wasn’t going to the pub…and I think there probably was an element of*,* “I’m not going to a football match…”*.

Another participant explained how he had gambled more during the initial lockdown to try to supplement his reduced income:

*08-2020-PG: I couldn’t make enough money*,* you know*,* to pay out my bills or*,* you know*,* to pay out all my daily expenses or*,* you know*,* my other living costs…so I thought to myself*,* what is another option…I’ll do this [gambling] and see if I can make a bit [of money]*.

Conversely, reductions in income, increases in outgoings or changes in spending priorities could led to decreased gambling:

*09-2020-MRG: I didn’t have the finances to do it [gamble]*,* because I was transferring from furlough [temporary paid leave] to starting to work again. So I just didn’t have the finances.*

#### Multifactorial influences on gambling behaviour during the pandemic

Taken together, evidence from the interviews suggests that it was common for a constellation of factors to influence gambling behaviour rather than a single factor. The constellation of factors could vary from person to person and at different times during the pandemic. Likewise, the same factor could have a different impact on the gambling behaviour of one person compared to another. Thus, respondents’ own narratives suggested that attributes, events and circumstances which were protective for one person could place another person on an apparently riskier trajectory.

## Discussion

Our longitudinal qualitative research has explored continuities and changes in gambling behaviour, during a period in which the supply of gambling was transformed in a kind of naturally occurring experiment, among a sample of regular sports bettors in Britain. The accounts, based on interviews carried out in 2020 and 2021, were characterised by flux and fluctuations in gambling behaviour [16]. It was common for gambling to increase during some periods of the pandemic and decrease during others, reflecting the dynamic and (often) challenging circumstances which people were living through at the time. Uptake of new gambling activities and increased participation in, online gambling were described, particularly in response to changes in the sports and gambling landscapes.

Our findings complement and extend an analysis of survey data collected from a different sample of sports bettors three months after the initial lockdown. Those results also highlight bidirectional changes in gambling behaviour [7]. For example, the survey data also showed that the pandemic precipitated profound changes in daily lives that were associated with changes in gambling behaviour, including increases in online gambling for some and product switching for others. Equally, it was clear that the initial periods of the pandemic (e.g. the first lockdown) provided an opportunity for some to reduce or stop their gambling, depending on their social, working, living and financial circumstances.

In addition to substantiating existing findings, results from our qualitative analysis complement and extend the limited evidence derived from other qualitative studies on gambling during the pandemic. Two studies report some similar findings: a small study based on eight interviews with gamblers in Britain in 2020, and a subsequent (2021) study [15], based on 30 interviews with people in Britain who had previously gambled with a credit card or had made, or wanted to make, a complaint about a gambling operator. These studies reported several reasons for gambling less during the early pandemic: the shutdown of gambling venues and major sports leagues; lack of enjoyment of online gambling and a reduction in social motivations and opportunities for gambling [14]. They also included financial issues (e.g. having less money, economic uncertainty and changes in spending priorities), self-reflection and changes in social context of gambling due to COVID-19 restrictions [15]. Reasons given by those who said they were gambling more during the pandemic included: the chance to win money, and exposure to gambling marketing [14]; the ease and availability of online gambling; and having more free time and fewer outgoings [15]. Both studies reported that participants cited boredom as a reason for gambling more.

The present study extends understandings of gambling during COVID-19, which have not been fully captured by existing cross-sectional studies of gambling (e.g. that have tended to focus on neutral or positive change at population level early in the pandemic). In contrast, our longitudinal study highlights that individual patterns of gambling were episodic, particularly as restrictions were eased and then reintroduced during the pandemic. Thus, the present study is consistent with earlier literature examining gambling careers, before the advent of COVID-19. Like these studies [16, 17], we identified the fluid nature of behaviour over time, and that continuities and changes in behaviour were influenced by a complex constellation of factors relating to individual circumstances and the broader social and environmental context. Our research also supports previous work [16] in highlighting that the same attributes, events and circumstances could have a different impact on the gambling behaviour of one person compared to another, leading to reductions or increases in levels of gambling and more or less risky practices. However, our research contributes new understanding of gambling in times of social disruption, in suggesting that ‘typical’ patterns of gambling behaviour (e.g. being episodic), and known risk and protective factors [6], have been amplified during the pandemic, leading in some cases to rapid and dynamic behaviour changes, particularly as restrictions were eased and then reintroduced.

Our findings have several implications for policy. First, findings illustrate a unique ‘natural experiment’ in terms of the provision of commercial gambling. National lockdowns created an unprecedented restriction of supply, whereby all land-based venues were closed and mainstream sports cancelled, dramatically reducing the availability of gambling throughout the world, including the UK. It is likely that this precipitated a move to online gambling practices in those who previously relied on land-based outlets [33]. Our findings illustrate and confirm the role of commercial actors (among other factors) in continuing to influence gambling participation and harms. Findings confirm the adaptability of the gambling industry itself and the speed and effectiveness with which industry can respond to changes in the gambling environment. Even in such unprecedented times, it rapidly developed a number of ways to circumvent commercial constraints by devising and promoting a range of alternative products, such as Belarusian football betting, e-sports betting and online casinos and games, and then seemingly redoubling its marketing efforts when live sport resumed [5]. These practices should be taken on board by policymakers and regulators, who should ensure that the industry is regulated by continual ‘horizon scanning’, to keep up to date with commercial developments and innovations and, if necessary, regulate accordingly.

Notably, we found limited evidence in this study of consumers turning to ‘black market’ products, even when regulated gambling opportunities were severely restricted early in the pandemic. This finding supports the need for caution around industry claims about the threat posed by the ‘black market’ in response to proposals for stricter gambling regulations [34]. Our findings, demonstrating nuance responses to change also challenges the current, and dominant, industry paradigm that further regulatory change will lead to a wholesale shift to unlicensed gambling provision. Our findings suggest that responses are likely to be much more varied that this.

Second, insights from our research on the influence of marketing on gambling behaviour may be of particular interest to policymakers in the UK who have judged there to be evidence gaps in this area, requiring further investigation [35], although this view has been challenged by some academics [36]. Our findings reinforce the reportedly harmful impact that gambling marketing and promotions can have on some people experiencing moderate risk or problem gambling (see also [28, 37]), and highlight the factors which appear to have made some people more susceptible to marketing during the pandemic. Some participants in our study were evidently distressed by the volume and nature of the direct marketing and promotions they received from operators and appeared to face difficulties in being able to limit exposures to direct (and other) marketing during this time, as has also been reported in pre-pandemic times [38]. Some participants’ accounts of the gambling marketing they received raise concerns that, at best, some gambling companies’ marketing techniques may not adequately differentiate between more or less vulnerable consumers, and at worst that some companies, including unregulated companies, may have exploited those most vulnerable during pandemic (see also [39]) Whichever is true, an increase in gambling among those experiencing risky or problem gambling is cause for concern and requires stronger measures to protect those who are currently inadequately protected from gambling marketing. Findings confirm weaknesses in current approaches to regulation of gambling marketing and promotional activity, which make it extremely difficult (perhaps almost impossible) for some people who are at risk of or experiencing harm to exercise ‘individual responsibility’.

Third, the episodic nature of individual gambling behaviour identified in this study reinforces the challenge for measuring the impact of the pandemic on gambling behaviour and estimating the scale of gambling harms experienced by gamblers and others affected by gambling during this time. It is thus inappropriate for policymakers to conclude that the pandemic had an overall neutral or positive impact on gambling based on cross-sectional studies conducted early in the pandemic.

Fourth, our results suggest that gambling behaviour continued to fluctuate during the pandemic, and could change quickly, in relation to a complex interplay of individual, relational, community and societal factors. This supports the need for a multifaceted and comprehensive regulatory approach to prevent and reduce gambling harms, with careful consideration given to wide ranging issues, including what products are available and their properties, how products are marketed to individuals, and provision of adequate services and support to respond to established and emergent harms. Interventions to facilitate and maintain positive behaviour change need to be flexible, free/affordable, and easy to access when people need them. Despite growing evidence on the harms of gambling, and on how consumers could be better protected from the industry, the UK Government’s 2023 White Paper on gambling reform has been criticised for failing to take a comprehensive public health approach to regulation [40, 41].

Our study has strengths and limitations. Novel features, which add value to the existing evidence base, centre on the use of longitudinal qualitative methods to provide rich insight into gambling behaviour and how and why this may have changed during the pandemic. Other strengths are that we collected data beyond the early months of the pandemic, and that data were collected in line with recognised criteria for robust qualitative research [30]. We also acknowledge some limitations. Our sample of sports bettors, although diverse, may not reflect the full range and diversity of views and experiences among sports bettors in Britain, particularly given the small number of women, non-white participants and gamblers experiencing low risk gambling in the sample. The collection of self-report data has limitations, but data on experience require these first-hand accounts. Nonetheless, such data may be subject to recall bias and some people may misperceive the influence of various factors on their own behaviour; for example, evidence suggests that people tend to believe that marketing has a greater impact on other people than on themselves [42]. Whilst most participants agreed to take part in a follow-up interview, it is possible that the three participants we could not recontact had different experiences. Several participants who took part in follow-up interviews, including some who were not experiencing gambling problems, told us that undertaking the first interview had promoted self-reflection which sometimes extended beyond the interview, and thus it is possible that their subsequent gambling behaviour may have been influenced in part by participation in the study. Further research is required to explore whether, and if so how, the pandemic has created long-term changes in gambling habits and the implications for gambling harms.

## Conclusion

This study adds novel and nuanced understanding of gambling trajectories during the COVID-19 pandemic. These trajectories were not linear, but rather characterised by fluctuations that were influenced by the different stages of the pandemic, as well as individual circumstances and the broader social and environmental contexts. The study sheds light on a unique ‘natural experiment’ in terms of the reduction of the supply of gambling due to COVID-19 restrictions. Despite the apparent commercial hiatus brought about by such restrictions, our study has highlighted the rapid adaptability of the gambling industry in terms of its ability to continue to find ways to reach, engage, and influence gamblers through myriad product offerings and markets. Findings from this study support the need for a multifaceted and comprehensive policy regulatory and treatment approach which considers product availability, product marketing (especially targeted marketing to individuals), consumer engagement and the provision of adequate support services that are flexible, responsive, and easy to access.

## Data Availability

The data that support the findings (grant number ES/V004549/1) of this study may be made available on request from the study PIs [KH, NC, HW], subject to participant consent and special conditions.

## References

[1] WHO. Mental Health and COVID-19: Early evidence of the pandemic’s impact WHO. 2022. Report No.: WHO/2019-nCoV/Sci_Brief/Mental_health/2022.1

[2] Sidik SM. How covid has deepened inequality — in six stark graphics: Nature; 2022. https://media.nature.com/original/magazine-assets/d41586-022-01647-6/d41586-022-01647-6.pdf 10.1038/d41586-022-01647-6 35732780

[3] Håkansson A, Fernández-Aranda F, Menchón JM, Potenza MN, Jiménez-Murcia S. Gambling During the COVID-19 Crisis – A Cause for Concern. J Addict Med. 2020;14(4). 10.1097/ADM.0000000000000690 PMC7273946 32433365

[4] van Schalkwyk M, Cheetham D, Reeves A, Petticrew M. Covid-19: we must take urgent action to avoid an increase in problem gambling and gambling related harms 2020. https://shorturl.at/XUfkT

[5] Critchlow N, Hunt K, Wardle H, Stead M. Expenditure on paid-for gambling advertising during the national COVID-19 ‘lockdowns’: an observational study of media monitoring data from the United Kingdom. J Gambl Stud. 2023;39(3):1451–65. 10.1007/s10899-022-10153-3. Epub 2022 Aug 29. PMID: 36031649; PMCID: PMC9420675. 10.1007/s10899-022-10153-3 PMC9420675 36031649

[6] Allami Y, Hodgins DC, Young M, Brunelle N, Currie S, Dufour M, et al. A meta-analysis of problem gambling risk factors in the general adult population. Addiction. 2021;116(11):2968–77. 33620735 10.1111/add.15449 PMC8518930

[7] Wardle H, Donnachie C, Critchlow N, Brown A, Bunn C, Dobbie F, et al. The impact of the initial Covid-19 lockdown upon regular sports bettors in Britain: Findings from a cross-sectional online study. Addict Behav. 2021;118:106876. 33647707 10.1016/j.addbeh.2021.106876 PMC9757982

[8] Williams L, MacDonald B, Rollins L, Janssen X, Fleming L, Grealy M, et al. Sharing positive behavior change made during COVID-19 lockdown: A mixed-methods coproduction study. Health Psychol. 2021;40(10):655–65. 34881933 10.1037/hea0001130

[9] Hodgins DC, Stevens RMG. The impact of COVID-19 on gambling and gambling disorder: emerging data. Curr Opin Psychiatry. 2021;34(4):332–43. 33859126 10.1097/YCO.0000000000000709 PMC8183251

[10] Brodeur M, Audette-Chapdelaine S, Savard A-C, Kairouz S. Gambling and the COVID-19 pandemic: A scoping review. Prog Neuropsychopharmacol Biol Psychiatry. 2021;111:110389. 34146652 10.1016/j.pnpbp.2021.110389 PMC8643271

[11] Sachdeva V, Sharma S, Sarangi A. Gambling behaviors during COVID-19: a narrative review. J Addict Dis. 2021;40(2):208–16. 10.1080/10550887.2021.1971942 10.1080/10550887.2021.1971942 34533420

[12] Quinn A, Grant JE, Chamberlain SR. COVID-19 and resultant restrictions on gambling behaviour. Neurosci Biobehavioral Reviews. 2022;143:104932. 10.1016/j.neubiorev.2022.104932 PMC9617674 36341942

[13] Public Health England. The impact of COVID-19 on gambling behaviour and gambling harms. A rapid review. London, UK: Public Health England; 2021.

[14] Gunstone B, Gosschalk K, Joyner O, Diaconu A, Sheikh M. The impact of the COVID-19 lockdown on gambling behaviour, harms and demand for treatment and support London: GambleAware; 2020. https://www.begambleaware.org/sites/default/files/2020-12/yougov-covid-19-report_0.pdf

[15] 2CV. Exploring the impact of Covid-19 on gambling behaviour: Gambling Commission 2021. https://www.gamblingcommission.gov.uk/statistics-and-research/publication/exploring-the-impact-of-covid-19-on-gambling-behaviour#key-facts

[16] Reith G, Dobbie F. Gambling careers: A longitudinal, qualitative study of gambling behaviour. Addict Res Theory. 2013;21(5):376–90.

[17] Kristiansen S, Reith G, Trabjerg CM. The notorious gambling class’: patterns of gambling among young people in Denmark. J Youth Stud. 2017;20(3):366–81.

[18] Rolando S, Beccaria F. Got to gamble, but I’ve got no money.’ A qualitative analysis of gambling careers in South Italy. Int Gambl Stud. 2019;19(1):106–24.

[19] Brown J, Kirk-Wade E, Coronavirus. A history of ‘lockdown laws’ in England London House of Commons Library 2021. https://researchbriefings.files.parliament.uk/documents/CBP-9068/CBP-9068.pdf

[20] SPICe Spotlight. Timeline of Coronavirus (COVID-19) in Scotland 2022. https://spice-spotlight.scot/2022/04/01/timeline-of-coronavirus-covid-19-in-scotland/

[21] Welsh Parliament Senedd Research. Coronavirus timeline: the response in Wales 2022. https://research.senedd.wales/research-articles/coronavirus-timeline-the-response-in-wales/

[22] BBC Sport. Coronavirus: How the virus has impacted sporting events around the world 2020. https://www.bbc.co.uk/sport/51605235

[23] Betting & Gaming Council. 10 Pledge Action Plan Announced. 2020. https://bettingandgamingcouncil.com/news/10pledges-safergambling/

[24] Betting & Gaming Council. BGC members to remove tv and radio gaming product advertising during covid-19 lockdown 2020. https://bettingandgamingcouncil.com/news/gaming-advertising-removed

[25] Purves RI, Maclean J, Rocha C, Philpott M, Fitzgerald N, Piggin J et al. Attending sporting mega events during COVID-19: mitigation and messaging at UK EURO 2020 matches. Health Promot Int. 2023;38(1). 10.1093/heapro/daac176 PMC9825819 36617291

[26] Hunt K, Critchlow N, Brown A, Bunn C, Dobbie F, Donnachie C, et al. Protocol for a Mixed-Method Investigation of the Impact of the COVID-19 Pandemic and Gambling Practices, Experiences and Marketing in the UK: The Betting and Gaming COVID-19 Impact Study. Int J Environ Res Public Health. 2020;17:22. 10.3390/ijerph17228449 PMC7697159 33203076

[27] Wardle H, Kolesnikov A, Fiedler I, Critchlow N, Hunt K. Is the economic model of gambling dependent on problem gambling? Evidence from an online survey of regular sports bettors in Britain. Int Gambl Stud. 2023;23(1):139–59.

[28] Wardle H, Critchlow N, Brown A, Donnachie C, Kolesnikov A, Hunt K. The association between gambling marketing and unplanned gambling spend: Synthesised findings from two online cross-sectional surveys. Addict Behav. 2022;135:107440. 35973384 10.1016/j.addbeh.2022.107440 PMC9587351

[29] Volberg RA, Williams RJ. Developing a Short Form of the PGSI. Report to the Gambling Commission. 2012. https://shorturl.at/A4FeN

[30] Ritchie J, Lewis J, McNaughton Nicholls C, Ormston R. Qualitative Research Practice London Sage; 2014.

[31] Gale NK, Heath G, Cameron E, Rashid S, Redwood S. Using the framework method for the analysis of qualitative data in multi-disciplinary health research. BMC Med Res Methodol. 2013;13(1):117. 24047204 10.1186/1471-2288-13-117 PMC3848812

[32] Samuelsson E, Wennberg P, Sundqvist K. Gamblers’ (mis-)interpretations of Problem Gambling Severity Index items: Ambiguities in qualitative accounts from the Swedish Longitudinal Gambling Study. Nordic Stud Alcohol Drugs. 2019;36(2):140–60. 10.1177/1455072519829407 PMC7434121 32934556

[33] Catalano A, Milani L, Franco M, Buscema F, Giommarini I, Sodano B, et al. The impact of COVID-19 pandemic on gambling: A systematic review. Addict Behav. 2024;155:108037. 38613856 10.1016/j.addbeh.2024.108037

[34] Wardle H, Reith G, Dobbie F, Rintoul A, Shiffman J. Regulatory Resistance? Narratives and Uses of Evidence around Black Market Provision of Gambling during the British Gambling Act Review. Int J Environ Res Public Health. 2021;18:21. 10.3390/ijerph182111566 PMC8582964 34770077

[35] Department for Culture MaS. High stakes: gambling reform for the digital age 2023 [ https://www.gov.uk/government/publications/high-stakes-gambling-reform-for-the-digital-age/high-stakes-gambling-reform-for-the-digital-age#chap2

[36] Newall P, Allami Y, Andrade M, Ayton P, Baker-Frampton R, Bennett D et al. ‘No evidence of harm’ implies no evidence of safety: Framing the lack of causal evidence in gambling advertising research. Addiction. 2023;n/a(n/a). 10.1111/add.16369 37953345

[37] McGrane E, Wardle H, Clowes M, Blank L, Pryce R, Field M, et al. What is the evidence that advertising policies could have an impact on gambling-related harms? A systematic umbrella review of the literature. Public Health. 2023;215:124–30. 36725155 10.1016/j.puhe.2022.11.019

[38] Syvertsen A, Pallesen S, Erevik EK, Mentzoni RA. Direct Marketing Experiences Among Individuals With Current and Lifetime Gambling Disorder. Front Psychol. 2020;11. 10.3389/fpsyg.2020.01957 PMC7419596 32849146

[39] Davies R, Kollewe J. William Hill to pay record £19.2m for ‘widespread and alarming’ failures: The Guardian;, ­­2023. [ https://shorturl.at/H6oEP

[40] Thomas SL, McKee M, Daube M. Gambling reform in the UK. BMJ. 2023;381:1026. 37160305 10.1136/bmj.p1026

[41] Iacobucci G. Gambling white paper: Doctors and campaigners lament lack of action on advertising. BMJ. 2023;381:p971. 10.1136/bmj.p971 37105577

[42] Newall PWS, Moodie C, Reith G, Stead M, Critchlow N, Morgan A, et al. Gambling Marketing from 2014 to 2018: a Literature Review. Curr Addict Rep. 2019;6(2):49–56.

